# Supplementary material for: Multi‐dataset identification of innovative feature genes and molecular mechanisms in keratoconus
Source: J Cell Mol Med. 2024 Sep 19;28(18):e70079. doi: 10.1111/jcmm.70079 (PMC11412914; doi:10.1111/jcmm.70079)
Supplement: Supplementary file 2 — Material S2. [file JCMM-28-e70079-s002.zip › 7.GSEA analysis/2.ATOH7/KEGG_CHEMOKINE_SIGNALING_PATHWAY.html]

Details for gene set KEGG\_CHEMOKINE\_SIGNALING\_PATHWAY[GSEA]

|  || Dataset | ATOH7.group.cls#h\_versus\_l |
| Phenotype | group.cls#h\_versus\_l |
| Upregulated in class | h |
| GeneSet | KEGG\_CHEMOKINE\_SIGNALING\_PATHWAY |
| Enrichment Score (ES) | 0.41264182 |
| Normalized Enrichment Score (NES) | 1.4497216 |
| Nominal p-value | 0.042226486 |
| FDR q-value | 0.27461356 |
| FWER p-Value | 0.849 |
Table: GSEA Results Summary

  

Fig 1: Enrichment plot: KEGG\_CHEMOKINE\_SIGNALING\_PATHWAY      
 Profile of the Running ES Score & Positions of GeneSet Members on the Rank Ordered List

  

| SYMBOL | TITLE | RANK IN GENE LIST | RANK METRIC SCORE | RUNNING ES | CORE ENRICHMENT || 1 | AKT3 | na | 9 | 1.043 | 0.0397 | Yes |
| 2 | CX3CL1 | na | 279 | 0.633 | 0.0562 | Yes |
| 3 | SHC2 | na | 290 | 0.628 | 0.0799 | Yes |
| 4 | GNB3 | na | 358 | 0.601 | 0.1010 | Yes |
| 5 | GNGT1 | na | 601 | 0.520 | 0.1139 | Yes |
| 6 | CXCL1 | na | 687 | 0.500 | 0.1306 | Yes |
| 7 | PRKACB | na | 829 | 0.474 | 0.1447 | Yes |
| 8 | CCR7 | na | 857 | 0.471 | 0.1619 | Yes |
| 9 | CXCL10 | na | 946 | 0.455 | 0.1768 | Yes |
| 10 | GNB4 | na | 955 | 0.454 | 0.1940 | Yes |
| 11 | CXCL3 | na | 964 | 0.453 | 0.2111 | Yes |
| 12 | CXCR1 | na | 1036 | 0.442 | 0.2260 | Yes |
| 13 | GNG11 | na | 1217 | 0.418 | 0.2368 | Yes |
| 14 | GRK7 | na | 1255 | 0.414 | 0.2516 | Yes |
| 15 | CXCL9 | na | 1354 | 0.402 | 0.2641 | Yes |
| 16 | CXCR4 | na | 1658 | 0.372 | 0.2696 | Yes |
| 17 | PLCB1 | na | 1718 | 0.367 | 0.2820 | Yes |
| 18 | VAV1 | na | 1743 | 0.365 | 0.2952 | Yes |
| 19 | PRKACG | na | 1784 | 0.361 | 0.3079 | Yes |
| 20 | PRKCB | na | 2281 | 0.324 | 0.3060 | Yes |
| 21 | CCL18 | na | 2431 | 0.314 | 0.3137 | Yes |
| 22 | CCL25 | na | 3272 | 0.266 | 0.2996 | Yes |
| 23 | XCL1 | na | 3325 | 0.264 | 0.3082 | Yes |
| 24 | DOCK2 | na | 3349 | 0.262 | 0.3176 | Yes |
| 25 | ELMO1 | na | 3456 | 0.258 | 0.3244 | Yes |
| 26 | CX3CR1 | na | 3478 | 0.257 | 0.3336 | Yes |
| 27 | CCL20 | na | 3493 | 0.256 | 0.3430 | Yes |
| 28 | CXCR3 | na | 3807 | 0.244 | 0.3433 | Yes |
| 29 | CCL7 | na | 4028 | 0.235 | 0.3459 | Yes |
| 30 | GNGT2 | na | 4165 | 0.230 | 0.3508 | Yes |
| 31 | MAP2K1 | na | 4240 | 0.228 | 0.3574 | Yes |
| 32 | GRK4 | na | 4315 | 0.225 | 0.3639 | Yes |
| 33 | CCL28 | na | 4434 | 0.220 | 0.3689 | Yes |
| 34 | PREX1 | na | 4826 | 0.207 | 0.3655 | Yes |
| 35 | CCL22 | na | 4847 | 0.206 | 0.3728 | Yes |
| 36 | CXCL8 | na | 4857 | 0.206 | 0.3804 | Yes |
| 37 | CCR9 | na | 4858 | 0.206 | 0.3883 | Yes |
| 38 | PAK1 | na | 4910 | 0.204 | 0.3946 | Yes |
| 39 | GRK1 | na | 5032 | 0.201 | 0.3988 | Yes |
| 40 | CXCL6 | na | 5077 | 0.199 | 0.4052 | Yes |
| 41 | ADCY3 | na | 5082 | 0.198 | 0.4126 | Yes |
| 42 | CRKL | na | 5867 | 0.175 | 0.3966 | No |
| 43 | PIK3R3 | na | 6166 | 0.166 | 0.3944 | No |
| 44 | RELA | na | 6248 | 0.164 | 0.3983 | No |
| 45 | LYN | na | 6447 | 0.158 | 0.3986 | No |
| 46 | GNG3 | na | 6965 | 0.144 | 0.3892 | No |
| 47 | NCF1 | na | 7020 | 0.143 | 0.3931 | No |
| 48 | CCR2 | na | 7444 | 0.132 | 0.3859 | No |
| 49 | TIAM2 | na | 7510 | 0.130 | 0.3890 | No |
| 50 | CCL5 | na | 7689 | 0.126 | 0.3887 | No |
| 51 | NRAS | na | 7693 | 0.126 | 0.3934 | No |
| 52 | CXCL12 | na | 7913 | 0.120 | 0.3917 | No |
| 53 | CCL19 | na | 7926 | 0.120 | 0.3959 | No |
| 54 | GRK5 | na | 8288 | 0.113 | 0.3898 | No |
| 55 | FGR | na | 8304 | 0.112 | 0.3936 | No |
| 56 | NFKB1 | na | 8680 | 0.105 | 0.3868 | No |
| 57 | CXCR6 | na | 8796 | 0.102 | 0.3874 | No |
| 58 | CCL14 | na | 8846 | 0.102 | 0.3899 | No |
| 59 | CCL8 | na | 8944 | 0.100 | 0.3909 | No |
| 60 | CRK | na | 8997 | 0.099 | 0.3932 | No |
| 61 | GNAI1 | na | 9135 | 0.096 | 0.3929 | No |
| 62 | WAS | na | 9234 | 0.095 | 0.3937 | No |
| 63 | CCL21 | na | 9273 | 0.094 | 0.3962 | No |
| 64 | GNB5 | na | 9551 | 0.089 | 0.3916 | No |
| 65 | GNG13 | na | 9586 | 0.089 | 0.3940 | No |
| 66 | GNG4 | na | 10174 | 0.079 | 0.3801 | No |
| 67 | JAK2 | na | 10386 | 0.075 | 0.3768 | No |
| 68 | GRB2 | na | 10441 | 0.075 | 0.3781 | No |
| 69 | ADCY8 | na | 10448 | 0.075 | 0.3808 | No |
| 70 | NFKBIA | na | 10503 | 0.073 | 0.3821 | No |
| 71 | PXN | na | 10526 | 0.073 | 0.3842 | No |
| 72 | SHC4 | na | 10680 | 0.071 | 0.3825 | No |
| 73 | CCL27 | na | 10842 | 0.068 | 0.3805 | No |
| 74 | PTK2 | na | 10929 | 0.067 | 0.3806 | No |
| 75 | CXCL16 | na | 11067 | 0.065 | 0.3791 | No |
| 76 | STAT3 | na | 11584 | 0.058 | 0.3664 | No |
| 77 | FOXO3 | na | 12029 | 0.052 | 0.3555 | No |
| 78 | XCR1 | na | 12064 | 0.052 | 0.3565 | No |
| 79 | GNB1 | na | 12091 | 0.051 | 0.3577 | No |
| 80 | PRKACA | na | 12347 | 0.048 | 0.3522 | No |
| 81 | GNG5 | na | 12533 | 0.046 | 0.3486 | No |
| 82 | PRKX | na | 12697 | 0.044 | 0.3455 | No |
| 83 | GNG12 | na | 12795 | 0.043 | 0.3443 | No |
| 84 | GNG10 | na | 12798 | 0.043 | 0.3459 | No |
| 85 | ARRB2 | na | 12937 | 0.041 | 0.3435 | No |
| 86 | GNG7 | na | 12945 | 0.041 | 0.3449 | No |
| 87 | NFKBIB | na | 13350 | 0.037 | 0.3346 | No |
| 88 | CXCL14 | na | 13489 | 0.035 | 0.3319 | No |
| 89 | RAC2 | na | 13916 | 0.031 | 0.3208 | No |
| 90 | CCL26 | na | 13929 | 0.031 | 0.3217 | No |
| 91 | CCL24 | na | 14065 | 0.030 | 0.3189 | No |
| 92 | CCL16 | na | 14253 | 0.028 | 0.3146 | No |
| 93 | GSK3A | na | 14426 | 0.026 | 0.3106 | No |
| 94 | MAPK1 | na | 14536 | 0.025 | 0.3084 | No |
| 95 | HRAS | na | 14559 | 0.025 | 0.3087 | No |
| 96 | SHC1 | na | 15160 | 0.020 | 0.2921 | No |
| 97 | RAF1 | na | 15219 | 0.019 | 0.2912 | No |
| 98 | RHOA | na | 15382 | 0.018 | 0.2872 | No |
| 99 | CCR1 | na | 15498 | 0.017 | 0.2845 | No |
| 100 | IKBKG | na | 15619 | 0.016 | 0.2816 | No |
| 101 | AKT1 | na | 15787 | 0.014 | 0.2773 | No |
| 102 | GSK3B | na | 16028 | 0.013 | 0.2709 | No |
| 103 | PIK3CD | na | 16202 | 0.011 | 0.2663 | No |
| 104 | RAC1 | na | 16331 | 0.010 | 0.2630 | No |
| 105 | CCL11 | na | 16481 | 0.009 | 0.2590 | No |
| 106 | CCL1 | na | 16556 | 0.008 | 0.2572 | No |
| 107 | MAPK3 | na | 16821 | 0.006 | 0.2498 | No |
| 108 | RAP1A | na | 16926 | 0.005 | 0.2470 | No |
| 109 | CCL23 | na | 17079 | 0.004 | 0.2427 | No |
| 110 | GRK6 | na | 17178 | 0.003 | 0.2400 | No |
| 111 | GNB2 | na | 17728 | -0.002 | 0.2242 | No |
| 112 | CCL3 | na | 17865 | -0.003 | 0.2203 | No |
| 113 | CDC42 | na | 17874 | -0.003 | 0.2202 | No |
| 114 | PRKCD | na | 17924 | -0.003 | 0.2189 | No |
| 115 | CXCL5 | na | 18023 | -0.004 | 0.2162 | No |
| 116 | RAP1B | na | 18052 | -0.005 | 0.2156 | No |
| 117 | WASL | na | 18186 | -0.006 | 0.2120 | No |
| 118 | STAT5B | na | 18496 | -0.008 | 0.2033 | No |
| 119 | CCR5 | na | 19311 | -0.016 | 0.1804 | No |
| 120 | ROCK2 | na | 19403 | -0.016 | 0.1784 | No |
| 121 | STAT2 | na | 19502 | -0.017 | 0.1762 | No |
| 122 | JAK3 | na | 19526 | -0.017 | 0.1762 | No |
| 123 | GNAI2 | na | 19897 | -0.021 | 0.1663 | No |
| 124 | TIAM1 | na | 19983 | -0.022 | 0.1647 | No |
| 125 | CCL2 | na | 20244 | -0.024 | 0.1580 | No |
| 126 | CHUK | na | 20245 | -0.024 | 0.1590 | No |
| 127 | PTK2B | na | 20285 | -0.025 | 0.1588 | No |
| 128 | PARD3 | na | 20314 | -0.025 | 0.1589 | No |
| 129 | CSK | na | 20669 | -0.028 | 0.1498 | No |
| 130 | PIK3CB | na | 20685 | -0.028 | 0.1504 | No |
| 131 | ADCY9 | na | 20701 | -0.029 | 0.1511 | No |
| 132 | PLCB4 | na | 20753 | -0.029 | 0.1507 | No |
| 133 | CCL17 | na | 20885 | -0.030 | 0.1481 | No |
| 134 | CXCL2 | na | 20917 | -0.031 | 0.1484 | No |
| 135 | VAV3 | na | 21180 | -0.034 | 0.1421 | No |
| 136 | PRKCZ | na | 21192 | -0.034 | 0.1431 | No |
| 137 | PIK3CA | na | 21359 | -0.036 | 0.1396 | No |
| 138 | ADCY2 | na | 21650 | -0.039 | 0.1327 | No |
| 139 | CCR4 | na | 21658 | -0.039 | 0.1340 | No |
| 140 | PF4V1 | na | 21861 | -0.041 | 0.1297 | No |
| 141 | VAV2 | na | 22149 | -0.045 | 0.1231 | No |
| 142 | PIK3R2 | na | 22209 | -0.045 | 0.1232 | No |
| 143 | SOS2 | na | 22550 | -0.049 | 0.1152 | No |
| 144 | SHC3 | na | 22782 | -0.052 | 0.1105 | No |
| 145 | CCL13 | na | 23352 | -0.060 | 0.0963 | No |
| 146 | IKBKB | na | 23356 | -0.060 | 0.0985 | No |
| 147 | STAT1 | na | 23384 | -0.060 | 0.1000 | No |
| 148 | PLCB3 | na | 24121 | -0.071 | 0.0815 | No |
| 149 | CXCL13 | na | 24658 | -0.080 | 0.0690 | No |
| 150 | GNG8 | na | 24911 | -0.084 | 0.0649 | No |
| 151 | BRAF | na | 25048 | -0.086 | 0.0643 | No |
| 152 | KRAS | na | 25083 | -0.087 | 0.0666 | No |
| 153 | CCL3L3 | na | 25086 | -0.087 | 0.0699 | No |
| 154 | ADCY5 | na | 25230 | -0.089 | 0.0692 | No |
| 155 | ADCY6 | na | 25322 | -0.091 | 0.0700 | No |
| 156 | CCR8 | na | 25351 | -0.091 | 0.0727 | No |
| 157 | ROCK1 | na | 25698 | -0.098 | 0.0665 | No |
| 158 | CXCR5 | na | 25913 | -0.102 | 0.0642 | No |
| 159 | PIK3CG | na | 26210 | -0.109 | 0.0598 | No |
| 160 | RASGRP2 | na | 26393 | -0.113 | 0.0588 | No |
| 161 | GNG2 | na | 26478 | -0.114 | 0.0608 | No |
| 162 | CCL15 | na | 26590 | -0.117 | 0.0620 | No |
| 163 | ADCY7 | na | 26708 | -0.119 | 0.0632 | No |
| 164 | SOS1 | na | 26852 | -0.122 | 0.0637 | No |
| 165 | PPBP | na | 26883 | -0.123 | 0.0676 | No |
| 166 | AKT2 | na | 27111 | -0.128 | 0.0659 | No |
| 167 | ADCY4 | na | 27391 | -0.136 | 0.0630 | No |
| 168 | HCK | na | 27450 | -0.137 | 0.0666 | No |
| 169 | CCR10 | na | 27510 | -0.139 | 0.0702 | No |
| 170 | CCR6 | na | 27670 | -0.142 | 0.0710 | No |
| 171 | PIK3R1 | na | 27720 | -0.144 | 0.0751 | No |
| 172 | CCR3 | na | 28176 | -0.156 | 0.0679 | No |
| 173 | GNAI3 | na | 28235 | -0.158 | 0.0723 | No |
| 174 | PLCB2 | na | 28336 | -0.160 | 0.0755 | No |
| 175 | BCAR1 | na | 28888 | -0.175 | 0.0663 | No |
| 176 | ITK | na | 29300 | -0.188 | 0.0616 | No |
| 177 | ARRB1 | na | 29554 | -0.196 | 0.0618 | No |
| 178 | CXCL11 | na | 29930 | -0.208 | 0.0589 | No |
| 179 | CXCR2 | na | 30340 | -0.223 | 0.0556 | No |
| 180 | ADCY1 | na | 30908 | -0.244 | 0.0485 | No |
| 181 | CCL4L2 | na | 33078 | -0.373 | 0.0000 | No |
| 182 | PIK3R5 | na | 34383 | -0.611 | -0.0143 | No |
| 183 | PF4 | na | 34429 | -0.634 | 0.0087 | No |
Table: GSEA details [plain text format]

  

Fig 2: KEGG\_CHEMOKINE\_SIGNALING\_PATHWAY      
 Blue-Pink O' Gram in the Space of the Analyzed GeneSet

  

Fig 3: KEGG\_CHEMOKINE\_SIGNALING\_PATHWAY: Random ES distribution      
 Gene set null distribution of ES for **KEGG\_CHEMOKINE\_SIGNALING\_PATHWAY**
